# Pre-immunization with an Intramuscular Injection of AAV9-Human Erythropoietin Vectors Reduces the Vector-Mediated Transduction following Re-Administration in Rat Brain

**DOI:** 10.1371/journal.pone.0063876

**Published:** 2013-05-08

**Authors:** Chun Yang, Wei-Hua Yang, Sha-Sha Chen, Bao-Feng Ma, Bin Li, Tao Lu, Ting-Yu Qu, Ronald L. Klein, Li-Ru Zhao, Wei-Ming Duan

**Affiliations:** 1 Department of Anatomy, Capital Medical University, Beijing, China; 2 Department of Cellular Biology and Anatomy, Louisiana State University Health Sciences Center, Shreveport, Lousiana, United States of America; 3 Department of Neurology, Louisiana State University Health Sciences Center, Shreveport, Lousiana, United States of America; 4 Department of Psychiatry, College of Medicine, University of Illinois at Chicago, Chicago, Illinois, United States of America; 5 Department of Pharmacology, Toxicology and Neuroscience, Louisiana State University Health Sciences Center, Shreveport, Lousiana, United States of America; 6 Department of Neurosurgery, Upstate Medical University, Syracuse, New York, United States of America; National Institute of Dental and Craniofacial Research, United States of America

## Abstract

We have recently demonstrated that adeno-associated virus serotype 9 (AAV9)-mediated human erythropoietin (hEPO) gene delivery into the brain protects dopaminergic (DA) neurons in the substantia nigra in a rat model of Parkinson's disease. In the present study, we examined whether pre-exposure to AAV9-hEPO vectors with an intramuscular or intrastriatal injection would reduce AAV9-mediated hEPO transduction in rat brain. We first characterized transgene expression and immune responses against AAV9-hEPO vectors in rat striatum at 4 days, 3 weeks and 6 months, and with doses ranging from 10^11^ to 10^13^ viral genomes. To sensitize immune system, rats received an injection of AAV9-hEPO into either the muscle or the left striatum, and then sequentially an injection of AAV9-hEPO into the right striatum 3 weeks later. We observed that transgene expression exhibited in a time course and dose dependent manner, and inflammatory and immune responses displayed in a time course manner. Intramuscular, but not intrastriatal injections of AAV9-hEPO resulted in reduced levels of hEPO transduction and increased levels of the major histocompatibility complex (MHC) class I and class II antigen expression in the striatum following AAV9-hEPO re-administration. There were infiltration of the cluster of differentiation 4 (CD4)-and CD8-lymphacytes, and accumulation of activated microglial cells and astrocytes in the virally injected striatum. In addition, the sera from the rats with intramuscular injections of AAV9-hEPO contained greater levels of antibodies against both AAV9 capsid protein and hEPO protein than the other treatment groups. hEPO gene expression was negatively correlated with the levels of circulating antibodies against AAV9 capsid protein. Intramuscular and intrastriatal re-administration of AAV9-hEPO led to increased numbers of red blood cells in peripheral blood. Our results suggest that pre-immunization with an intramuscular injection can lead to the reduction of transgene expression in the striatal re-administration.

## Introduction

Adeno-associated virus (AAV) vectors show promise for gene therapy of chronic neurological disorders including Parkinson's disease (PD) [1], [2], due to their non-pathogenic, ability to transduce non-dividing cells and dividing cells, long-term transgene expression [3], [4], no detected toxicity and minimal immune responses in transduced regions [4], [5]. Numerous studies have demonstrated that recombinant AAV vectors with newer serotypes have improved gene transfer into the rodent brain [6]–[10]. AAV9 vectors are of particular interest because they are capable of passing through the blood-brain barrier (BBB) and transducing both glial and neuronal cells in the brain and spinal cord [7]–[11]. We have recently shown that intrastriatal injections of AAV9 carrying a human erythropoietin (hEPO) gene result in a robust hEPO transduction in the striatum and protect nigral DA neurons from 6-hydroxydopamine (6-OHDA) toxicity in a rat model of PD, suggesting its therapeutic potential for PD [12]. However, immune responses against AAV9-hEPO vectors in rat brain have not yet been determined. Clinical studies have shown that the translations of AAV-mediated gene therapy into humans unexpectedly result in only short-term expression of the therapeutic. It suggests that an immune response against AAV vectors plays a very important role in the obstacle for successful translations to humans. Indeed, a significant portion of human population has been found to preexist neutralizing antibodies to the AAV [13], [14], and to present AAV capsid-specific T cells [15]. The AAV-mediated transgene expression may be precluded because of the preexisting neutralizing antibodies and AAV capsid-specific T cells.

There has been substantial evidence showing that AAV-mediated gene delivery can trigger humoral and cellular immune responses [16]–[26]. As one significant obstacle to AAV-based gene therapy is the high prevalence of neutralizing antibodies in humans, animal studies have demonstrated that prevention effects of in vivo AAV transduction of neutralizing antibodies are serotype specific and in a dose dependent manner [21], [23]. Rapti et al. also showed that low levels of neutralizing antibody prevented in vivo transduction by AAV9 in rats [24]. In a recent study, an intracerebral injection of AAV9-human aromatic L-amino acid decarboxylase (hAADC) has been shown to transduce antigen presenting cells (APCs) in the brain and to provoke a full immune response [17]. Interestingly, it has been demonstrated that induction of immunity to antigens expressed by recombinant AAV depends on the route of administration [16], [18]. When AAV-ovalbumin was administered intraperitoneally, intravenously or subcutaneously, mice developed ovalbumin-specific cytotoxic T cells (CTLs), anti-ovalbumin antibodies and antibodies to AAV. In contrast, when AAV-ovalbumin was administered intramuscularly, mice developed a humoral response to the virus and the transgene but minimal ovalbumin-specific CTLs. Besides the humoral and cellular immunity, complement system has also been found to be an essential component of the host immune response to AAV [27]. It has been shown that transient immunosuppression allows successful re-administration of AAV vectors in peripheral sites in mice, suggesting that immune response plays an important role in the stability of AAV-mediated transgene expression [28]–[30].

As AAV-mediated therapeutic gene delivery into the brain for chronic neurological disorders, such as PD, may necessitate re-administration of viral vectors in order to reach a desired therapeutic level of transgene products for long-term, several studies have examined ability of striatal re-administration of AAV vectors [21], [22], [31], [32]. Striatal re-administration of AAV vectors has yielded conflicting results. Some studies have shown that inflammatory and immune responses generated after the first administration in the brain prevent or inhibit transduction following the second administration, and some have demonstrated that successful re-administration in the brain may be possible. Discrepancies in the results may be due in part to methods of viral administration and possible transgene products. Providing the nature of the brain as an immunologically privileged site, studies of striatal re-administration of AAV vectors may provide insight into the basic brain immune response to AAV and also an experimental basis for potential translational applications of gene therapy for neurological disorders.

In a recent study, we developed an efficient AAV9-hEPO gene transfer system resulting in a robust and long-term hEPO transgene expression in the striatum of 6-OHDA-lesioned rats [12]. hEPO was found to be very stable in rat brain, and the hEPO transgene expression in the striatum was very easily immunocytochemically quantified. We thought that the effects of local inflammatory and immune responses on AAV-mediated transgene expression in rat brain should be characterized as measured by hEPO transgene products. In the present study, we therefore used this model system to systematically examine inflammatory and immune responses against AAV9-hEPO in the brain of immunized rats with different routes of AAV administration. We first examined the temporal and dose dependent patterns of inflammatory and immune responses against AAV9-hEPO in the rat brain. We then immunized rats with different routes of AAV9-hEPO administration (intrastriatal or intramuscular injections), and compared the outcome of AAV9-hEPO transgene expression, and the nature of inflammatory and immune responses against AAV9-hEPO in rat brain following re-administration. In addition, we examined the factors responsible for the elimination of AAV9-mediated hEPO transgene expression. We attempted to address the following issues: 1) whether the first injections of AAV9-hEPO into the left striatum could immunize rats and therefore jeopardize AAV9-mediated hEPO transgene expression in the right striatum following re-administration. 2) whether intramuscular injections of AAV9-hEPO could efficiently immunize rats, leading to reduction or elimination of AAV9-mediated hEPO transgene expression in the right striatum following re-administration. Of all species tested, naïve rats displayed the lowest levels of neutralization antibodies against AAV [24]. The use of naïve rats therefore allows us to mimic immunization status of patients who pre-exist neutralizing antibodies to the AAV and present AAV capsid-specific T cells, and to address clinical related issues whether this primed immunization status could reduce or prohibit AAV-mediated transgene expression.

## Results

### AAV9-mediated hEPO transduction in the striatum

**Figure 1:**
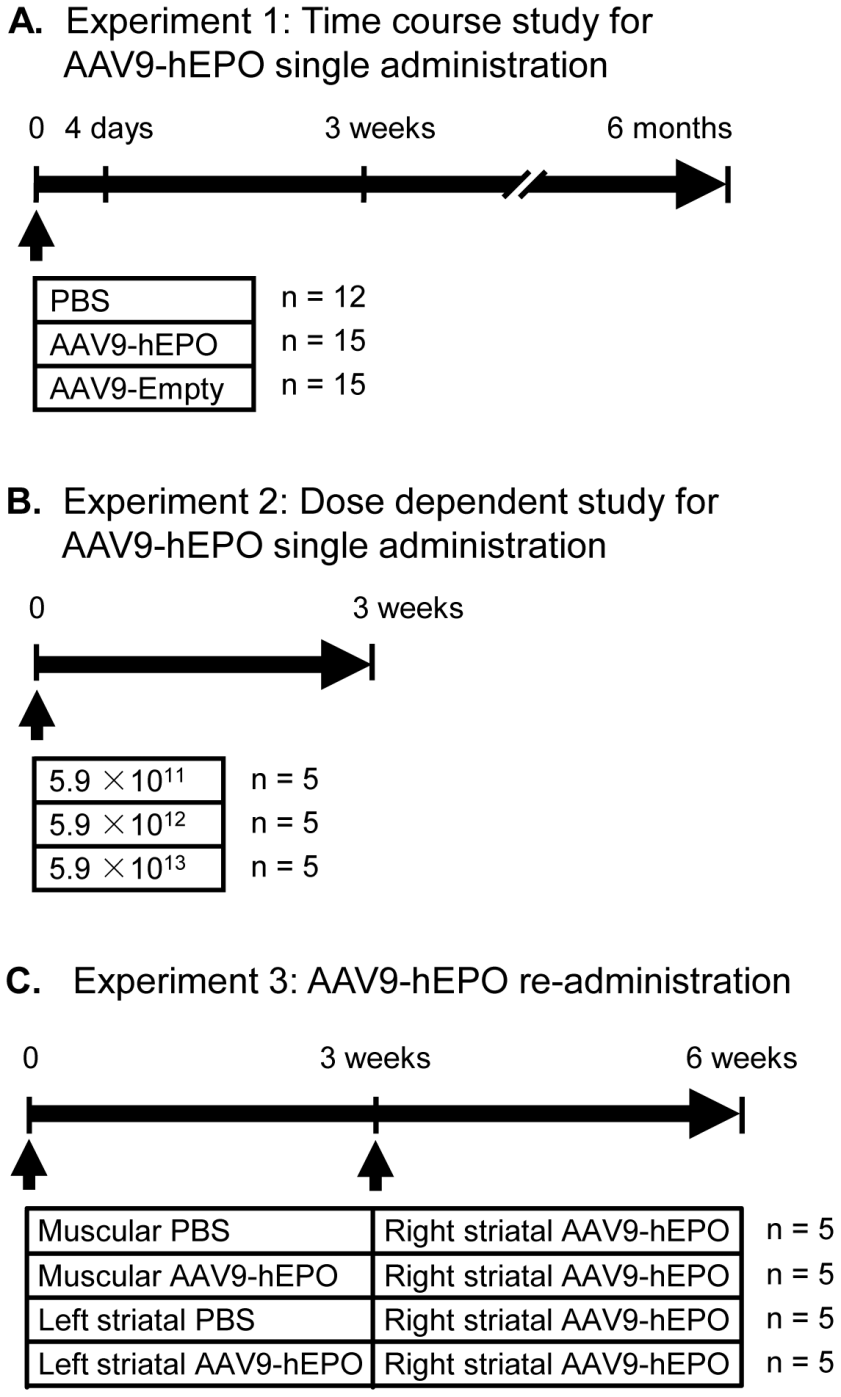
Experimental design. The timing and experimental groups are schematically illustrated for each experiment in the study. The number of rats is indicated for each treatment group at the right of their treatment regimen schematic. (A) Experiment 1: Time course study of recombinant adeno-associated virus serotype 9-human erythropoietin (AAV9-hEPO) single administration. Rats were assigned into three groups and received injections of 2 µl of sterile PBS, AAV9-hEPO (5.9×10^13^ vg ml^−1^) or AAV9-Empty (5.9×10^13^ vg ml^−1^) into the right striatum. Rats in each treated group were further divided into three subgroups and sacrificed at three time points (4 days, 3 weeks and 6 months after intrastriatal injections). Rat brains were immunocytochemically processed for histological evaluation. (B) Experiment 2: Dose dependent study of AAV9-hEPO single administration. Rats were assigned into three groups and received injections of 2 µl of AAV9-hEPO at three different titers (5.9×10^11^ vg ml^−1^, 5.9×10^12^ vg ml^−1^, or 5.9×10^13^ vg ml^−1^) into the right striatum. After 3 weeks, rats were sacrificed and rat brains were immunocytochemically processed for histological evaluation. (C) Experiment 3: AAV9-hEPO re-administration study. Rats were assigned into four groups: Group 1 (denoted as M PBS-Str hEPO), Rats received the first injections of 200 µl of sterile PBS into the right quadriceps, and the second injections of 2 µl of AAV9-hEPO (5.9×10^13^ vg ml^−1^) into the right striatum 3 weeks later; Group 2 (M hEPO-Str hEPO), Rats received the first injections of 200 µl of AAV9-hEPO (5.9×10^11^ vg ml^−1^) into the right quadriceps, and the second injections of 2 µl of AAV9-hEPO (5.9×10^13^ vg ml^−1^) into the right striatum 3 weeks later; Group 3 (Str PBS-Str hEPO), Rats received the first injections of 2 µl of sterile PBS into the left striatum, and the second injections of 2 µl of AAV9-hEPO (5.9×10^13^ vg ml^−1^) into the right striatum 3 weeks later; and group 4 (Str hEPO-Str hEPO), Rats received the first injections of 2 µl AAV9-hEPO (5.9×10^13^ vg ml^−1^) into the left striatum, and the second injections of 2 µl of AAV9-hEPO (5.9×10^13^ vg ml^−1^) into the right striatum 3 weeks later. Peripheral blood samples were collected prior to sacrifice of rats for hematological, and antibody and immunoneutralization assays, and rat brains were immunocytochemically processed for histological evaluation 3 weeks after the second injections. Arrow heads indicate the time points of injections. M, muscular; Str, striatal.

AAV9-mediated hEPO transgene expression was examined in the time course (Fig. 1A) and dose-dependent (Fig. 1B) studies for AAV9-hEPO single administration, and in the study for AAV9-hEPO re-administration (Fig. 1C).

**Figure 2:**
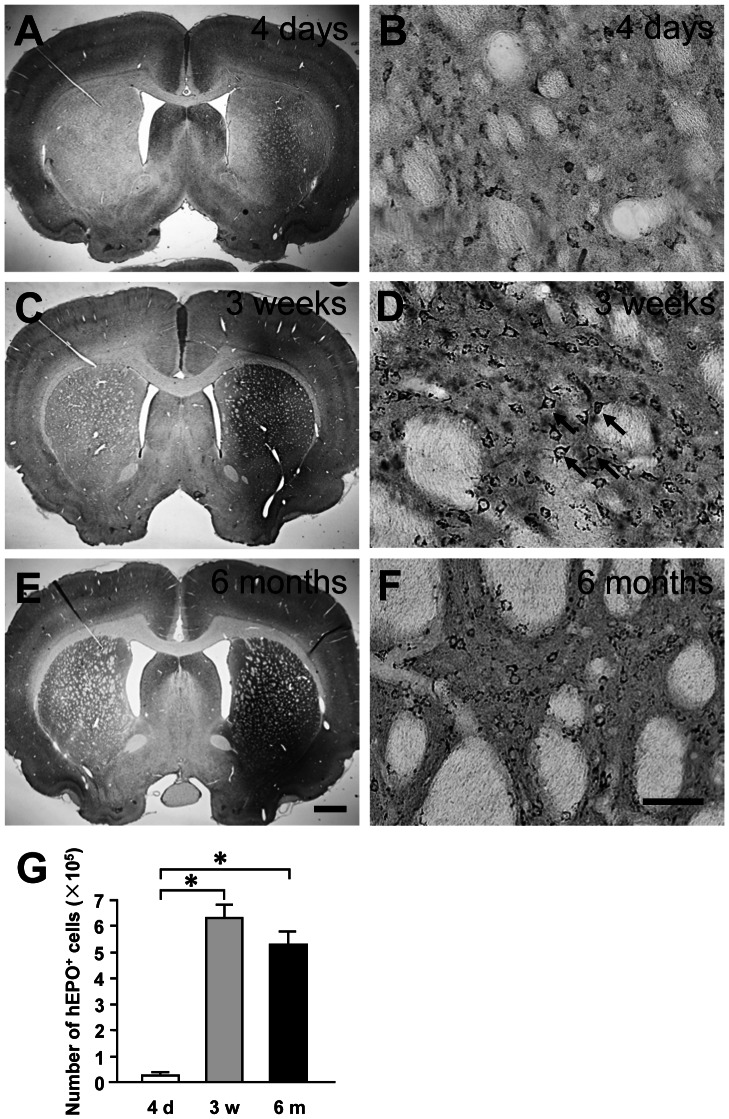
Temporal expression of transgene (human erythropoietin, hEPO) in the rat brain after intrastriatal injections of recombinant adeno-associated virus serotype 9-hEPO (AAV9-hEPO). Photomicrographs were prepared from coronal sections through the striatum and processed for hEPO immunocytochemistry from representative rats 4 days (A and B), 3 weeks (C and D) and 6 months (E and F) after intrastriatal injections of 2 µl of AAV9-hEPO (5.9×10^13^ vg ml^−1^). B, D and F are high magnification photomicrographs for A, C and D, respectively. The scale bar = 1 mm in E also for A and C; 50 µm in F also for B and D. The arrows (D) indicate representative hEPO-immunoreactive (IR) cells. (G) Stereological quantification of hEPO-IR cells in the injected striatum of rats 4 days (open bar, n = 5), 3 weeks (gray bar, n = 5) and 6 months (black bar, n = 4) after injections. The data are presented as mean values±standard error of the mean (SEM). A one-factor analysis of variance (ANOVA) followed by Fisher's *post hoc* test was applied to compare hEPO-IR cell counts between different time points. * p<0.05 versus 4 days group.

To examine the time course of human hEPO transgene expression in the virally injected striatum, rats received single injections of 2 µl AAV9-hEPO (5.9×10^13^ vg ml^−1^) into the right striatum. As controls, rats received single injections of 2 µl phosphate buffered saline (PBS) or 2 µl AAV9-Empty (5.9×10^13^ vg ml^−1^) into the right striatum. After 4 days, 3 weeks and 6 months, rats were sacrificed and the brains were immunocytochemically processed for hEPO transgene product. At 4 days, immunocytochemical staining showed that AAV9-mediated hEPO gene transduction in the injected striatum was at a low level. hEPO immunostaining was light in the injected striatum, and hEPO-immunoreactive (IR) cells were barely detected in low magnification images of striatal sections (Fig. 2A). High magnification photomicrographs showed that hEPO-IR cells resembled striatal medium-spiny cells with a few processes in the striatum (Fig. 2B). At 3 weeks, the levels of AAV9-mediated hEPO gene transduction were significantly increased in the injected striatum (Fig. 2C and D). hEPO immunostaining was intensive and hEPO-IR cells were distributed throughout in a large area of the striatum. At 6 months, the pattern of hEPO transduction was similar to that seen 3 weeks after viral injections. hEPO immunostaining remained intensive and hEPO-IR cells were distributed throughout in a large area of the striatum (Fig. 2E and F). Unbiased stereological cell population estimation showed that the total numbers of hEPO-IR cells in the injected striatum were 24599±14304 at 4 days, 629222±50775 at 3 weeks and 526463±51188 at 6 months. The total numbers of hEPO-IR cells at both 3 weeks and 6 months were significantly greater than that at 4 days (* p<0.05) (Fig. 2G). There was no significant difference in the number of hEPO-IR cells in the injected striatum between 3- week and 6-month time-points (p>0.05).

**Figure 3:**
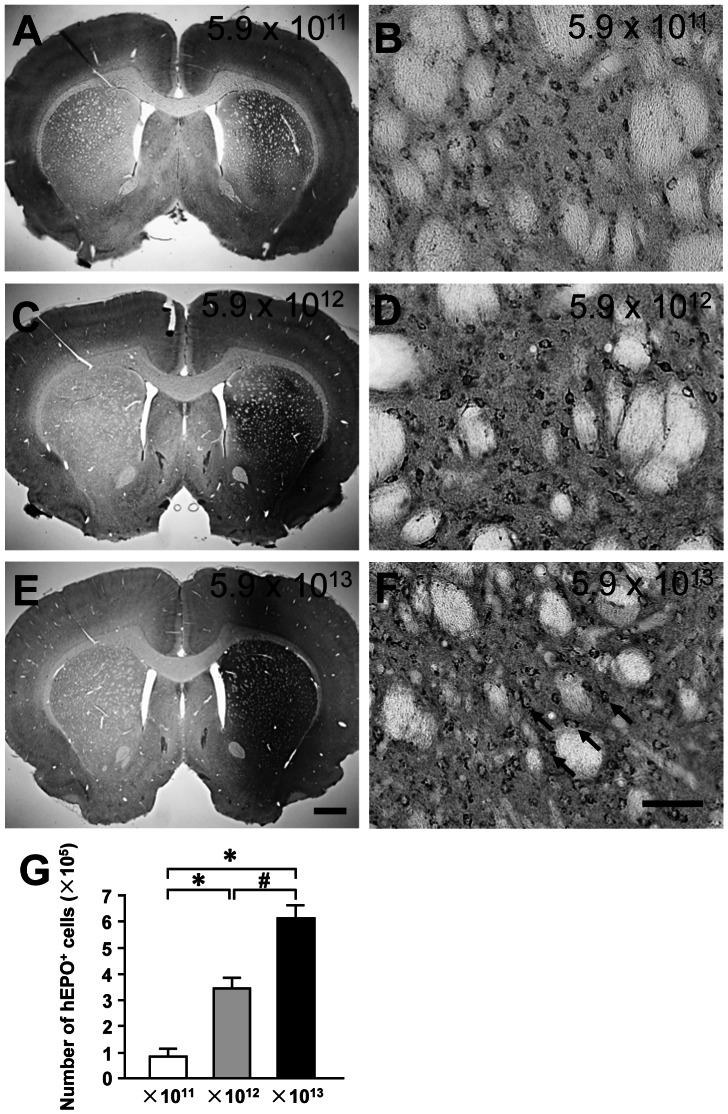
Dose dependent expression of transgene (human erythropoietin, hEPO) in the rat brain after intrastriatal injections of recombinant adeno-associated virus serotype 9-hEPO (AAV9-hEPO). Photomicrographs were prepared from coronal sections through the striatum and processed for hEPO immunocytochemistry from representative rats received intrastriatal injections of 2 µl of AAV9-hEPO with a titer of 5.9×10^11^ vg ml^−1^ (A and B), 5.9×10^12^ vg ml^−1^ (C and D) or 5.9×10^13^ vg ml^−1^ (E and F) 3 weeks after injection. B, D and F are high magnification photomicrographs for A, C and E, respectively. The scale bar = 1 mm in E also for A and C; 50 µm in F also for B and D. The arrows (F) indicate representative hEPO-immunoreactive (IR) cells. (G) Stereological quantification of hEPO-IR cells in the injected striatum of rats received intrastriatal injections of 2 µl of AAV9-hEPO with a titer of 5.9×10^11^ vg ml^−1^ (open bar, n = 5), 5.9×10^12^ vg ml^−1^ (gray bar, n = 5) or 5.9×10^13^ vg ml^−1^ (black bar, n = 5) 3 weeks after injection. The data are presented as mean values±SEM. A one-factor analysis of variance (ANOVA) followed by Fisher's *post hoc* test was applied to compare hEPO-IR cell counts between different titer groups. * p<0.05 versus 5.9×10^11^ group. # p<0.05 versus the indicated groups.

To examine the dose-dependent hEPO transgene expression in the striatum, rats received single injections of 2 µl AAV9-hEPO at three different titers (5.9×10^11^ vg ml^−1^, 5.9×10^12^ vg ml^−1^, or 5.9×10^13^ vg ml^−1^) into the right striatum. After 3 weeks, rats were sacrificed and the brains were immunocytochemically processed for hEPO transgene product. hEPO immunostaining showed that a low dose of AAV9-hEPO (2 µl, 5.9×10^11^ vg ml^−1^) resulted in low levels of hEPO gene transduction in the injected striatum. hEPO immunostaining was light in the injected striatum, and hEPO-IR cells were barely detected in low magnification images of striatal sections (Fig. 3A). High magnification photomicrographs showed that hEPO-IR cells resembled striatal medium-spiny cells with a few processes in the striatum (Fig. 3B). An intermediate dose of AAV9-hEPO (2 µl, 5.9×10^12^ vg ml^−1^) led to significantly increased levels of AAV9-mediated hEPO gene transduction in the injected striatum (Fig. 3C and D). hEPO immunostaining was intensive and hEPO-IR cells were distributed throughout in a large area of the striatum. A high dose of AAV9-hEPO (2 µl, 5.9×10^13^ vg ml^−1^) further significantly increased levels of hEPO transduction in the injected striatum. hEPO immunostaining appeared to be more intensive and hEPO-IR cells were distributed throughout in a large area of the striatum (Fig. 3E and F). Unbiased stereological cell population estimation showed that the numbers of hEPO-IR cells in the injected striatum were 81231±30005 for the low dose group, 341305±43338 for the intermediate dose group, and 610481±53834 for the high dose group (Fig. 3G). The total number of hEPO-IR cells in the intermediate dose group was significantly greater than that in the low dose group (* p<0.05). The total number of hEPO-IR cells in the high dose group was significantly greater than those in other two doses groups (*, # p<0.05). These data suggest that AAV9-mediated hEPO transduction in the striatum is in a good dose-dependent manner.

**Figure 4:**
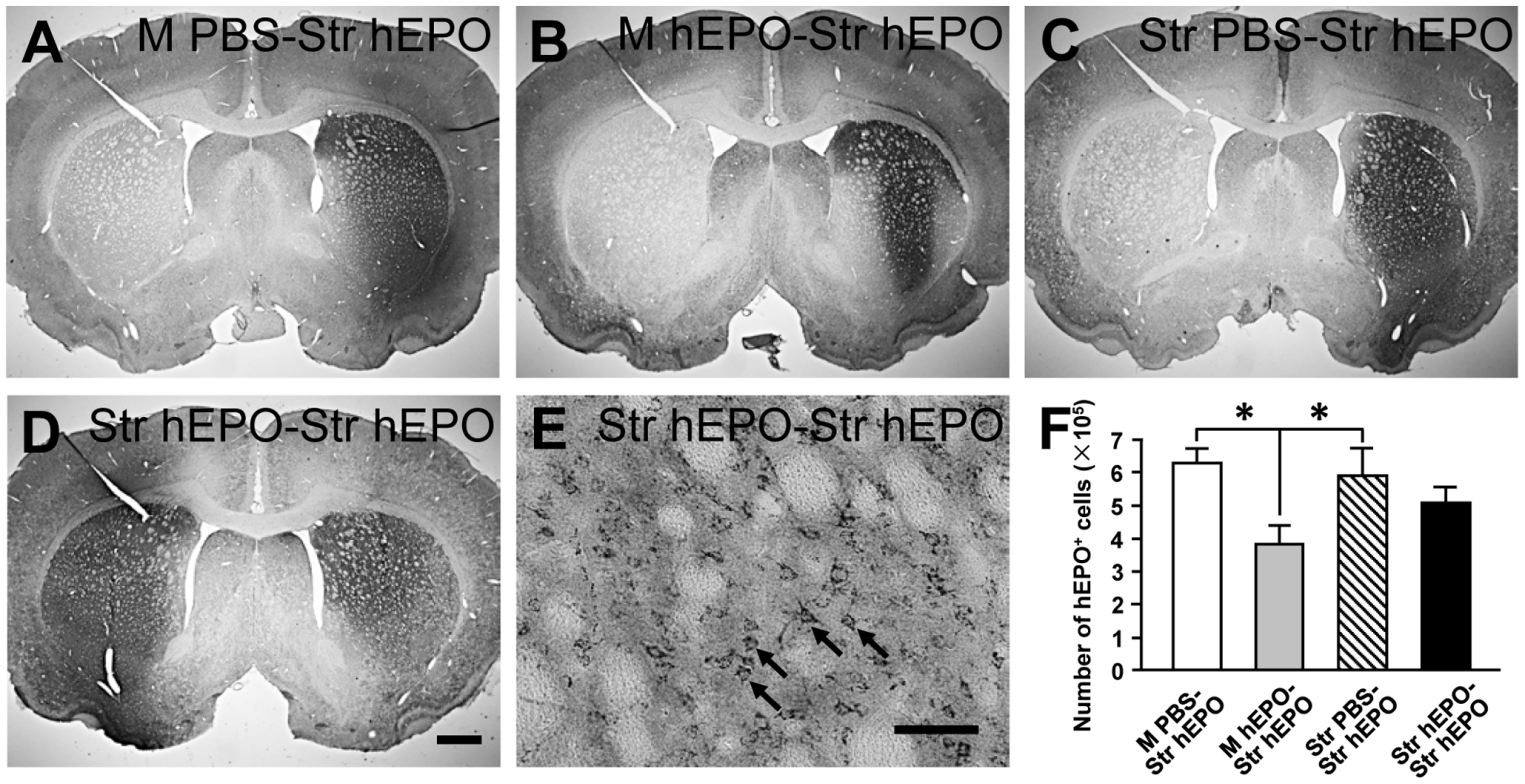
Transgene (human erythropoietin, hEPO) expression in the rat brain after intrastriatal injections of recombinant adeno-associated virus serotype 9-hEPO (AAV9-hEPO) from the M PBS-Str hEPO (A), M hEPO-Str hEPO (B), Str PBS-Str hEPO (C), and Str hEPO-Str hEPO (D and E) groups. Rats first received intramuscular (200 µl, 5.9×10^11^ vector genomes, vg ml^−1^) or intrastriatal (2 µl, 5.9×10^13^ vector genomes, vg ml^−1^) injections for 3 weeks and then received AAV9-hEPO re-administration into the right striatum (2 µl, 5.9×10^13^ vector genomes, vg ml^−1^). Rats were sacrificed 3 weeks after AAV9-hEPO re-administration. Photomicrographs were prepared from coronal sections through the striatum and processed for hEPO immunocytochemistry. (E) is a high magnification photomicrograph of the right injected striatum for (D). The scale bar = 1 mm in D also for A, B and C; 50 µm for E. The arrows (E) indicate representative hEPO-immunoreactive (IR) cells. (F) Stereological quantification of hEPO-IR cells in the right injected striatum of rats from the M PBS-Str hEPO (open bar, n = 5), M hEPO-Str hEPO (gray bar, n = 5), Str PBS-Str hEPO (hatched bar, n = 5), and Str hEPO-Str hEPO (black bar, n = 5) groups 3 weeks after the second injections. The data are presented as mean values ± SEM. A one-factor analysis of variance (ANOVA) followed by Fisher's *post hoc* test was applied to compare hEPO-IR cell counts between different groups. * p<0.05 versus the M hEPO-Str hEPO group.

To examine effects of immunization in the different status on AAV9-mediated hEPO transduction in the striatum, we designed the AAV9-hEPO re-administration experiment. Rats were immunized by intramuscular (200 µl, 5.9×10^11^ vector genomes, vg ml^−1^) or intrastriatal (2 µl, 5.9×10^13^ vector genomes, vg ml^−1^) approaches for 3 weeks and then received AAV9-hEPO re-administration into the right striatum (2 µl, 5.9×10^13^ vector genomes, vg ml^−1^). Rats were sacrificed 3 weeks after AAV9-hEPO re-administration, and the brains were immunocytochemically processed for hEPO transgene product. hEPO immunostaining showed that AAV9-mediated hEPO transduction was robust and widespread in the injected striatum of rats in the M PBS-Str hEPO, Str PBS-Str hEPO and Str hEPO-Str hEPO groups (Fig. 4A, C, D, and E). hEPO immunostaining was intensive and hEPO-IR cells were distributed throughout in a large area of the striatum. High magnification photomicrographs showed that hEPO-IR cells resembled striatal medium-spiny cells with a few processes in the striatum (Fig. 4E). In contrast AAV9-mediated hEPO transduction was localized around the needle track in the injected striatum of rats in the M hEPO-Str hEPO group (Fig. 4B). Unbiased stereological cell population estimation showed that the total numbers of hEPO-IR in the injected striatum were 630168±49820 for the M PBS-Str hEPO group, 601780±78766 for the Str PBS-Str hEPO group and 510015±51876 for the Str hEPO-Str hEPO group (Fig. 4F). Although the number of hEPO-IR cells in the injected striatum appeared to be lower in the Str hEPO-Str hEPO group than that in the M PBS-Str hEPO and Str PBS-Str hEPO groups and to be higher than that in the M hEPO-Str hEPO group, the differences did not reach to a significant level (p>0.05). However, the total number of hEPO-IR cells in the injected striatum was significantly reduced to 381673±63253 in the M hEPO-Str hEPO group when compared to the M PBS-Str hEPO and Str PBS-Str hEPO groups (Fig. 4F, * p<0.05). These data suggest that immunization with intramuscular administration of AAV9-hEPO can significantly reduce the same type of viruses, AAV9-mediated hEPO transduction in the brain after striatal re-administration. However, the effect of intrastriatal administration of AAV9-hEPO on AAV9-mediated hEPO transduction after striatal re-administration is minimal in the current animal model system.

### Inflammatory and immune responses in the virally-injected striatum

Similar to examination of AAV9-mediated hEPO transgene expression in the injected striatum, inflammatory and immune responses in the virally-injected striatum were also evaluated in the time course and dose-dependent studies for AAV9-hEPO single administration, and in the study for AAV9-hEPO re-administration.

The time course of inflammatory and immune responses in the injected striatum was examined by using the major histocompatibility complex (MHC) class I and class II, cluster of differentiation (CD)4, CD8, complement receptor (CR)3 and glial fibrillary acidic protein (GFAP) immunostaining. In general, there was a similar temporal pattern of host inflammatory and immune responses in the striatum of rats received a single administration of PBS, AAV9-hEPO, or AAV9-Empty. During the course of the experiment, one rat died in the single administration of AAV9-hEPO group for 6-month time-point subgroup, and was therefore not included in the histological analyses.

**Figure 5:**
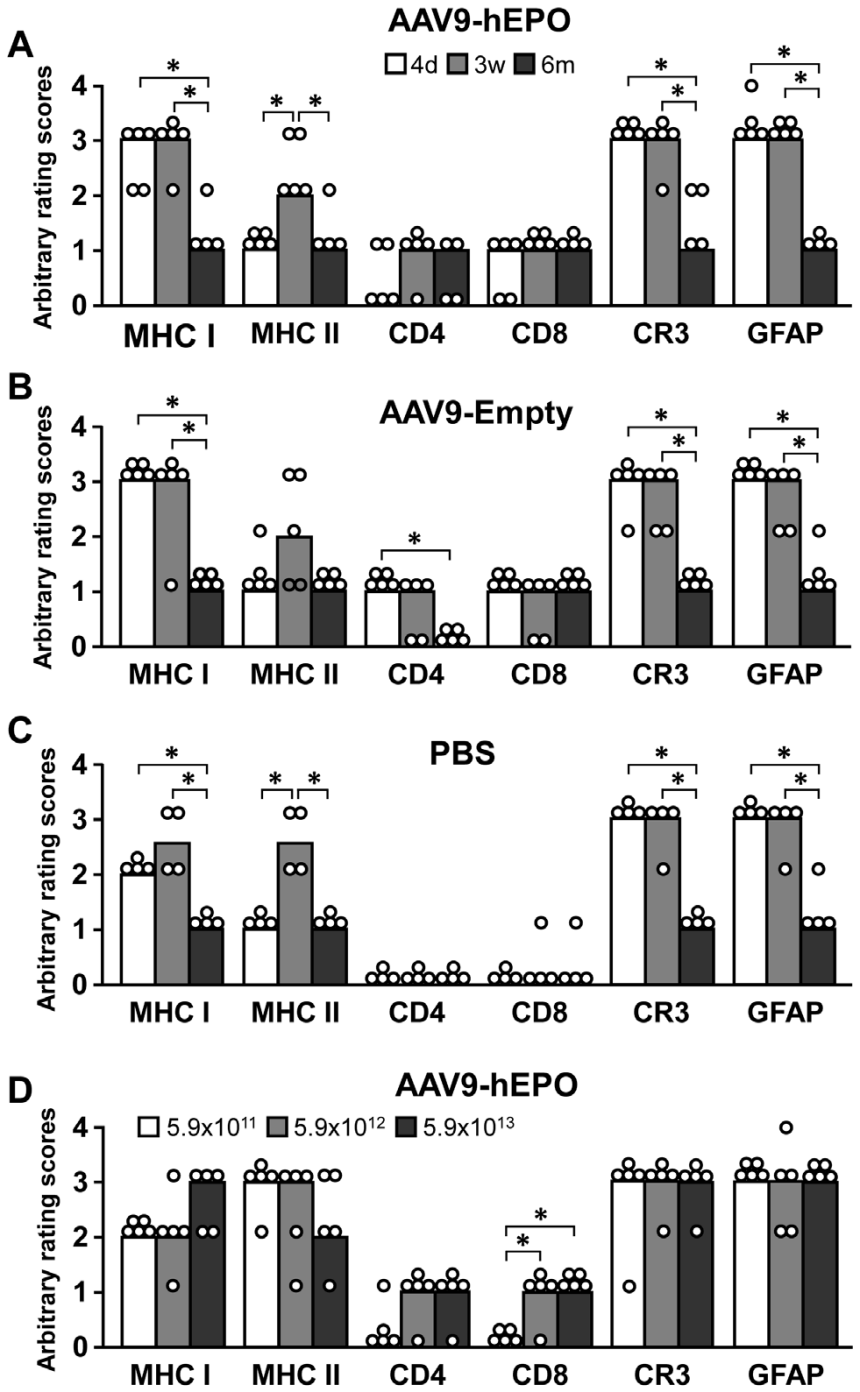
Arbitrary rating scores of major histocompatibility complex (MHC) class I, MHC class II, cluster of differentiation 4 (CD4), CD8, complement receptors 3 (CR3) and glial fibrillary acidic protein (GFAP) immunostaining in the right striatum of rats received 2 µl of recombinant adeno-associated virus serotype 9-human erythropoietin (AAV9-hEPO, 5.9×10^13^ vg ml^−1^) (A), AAV9-Empy (5.9×10^13^ vg ml^−1^) (B), or sterile phosphate buffered saline (PBS) (C) at 4 days (open bars, n = 4), 3 weeks (gray bars, n = 5) and 6 months (black bars, n = 5) time points, and of rats received 2 µl of AAV9-hEPO at a titer of 5.9×10^11^ vg ml^−1^ (open bars, n = 5), 5.9×10^12^ vg ml^−1^ (gray bars, n = 5), or 5.9×10^13^ vg ml^−1^ (black bars, n = 5) 3 weeks after injections (D). The bars represent the median values of rating scores, and the circles depict the individual values. A Kruskal-Wallis test followed by Mann-Whitney U tests was used to compare the rating scores between groups. * p<0.05 versus indicated groups.

For inflammatory and innate immune responses, we used CR3 and GFAP antibodies to examine microglial and astrocytic reaction in the striatum, respectively. Cells immunoreactive for CR3 resembled either typical microglia or macrophages. Both resting and activated microglia were stained by the antibody. Microglia which had large perikarya and were stained more intensely were considered as activated microglia. Both resting and activated astrocytes were also stained by the GFAP antibody. Resting astrocytes had a stellar shape with fine processes and were found in both injected and contralateral striatum. Activated astrocytes were intensely immunostained and had large perikarya and thick processes. They were mainly located in the injection sites. Figure 5A, B, and C show arbitrary ratings of CR3 and GFAP immunostaining in the injected striata containing the needle tracks. There were large numbers of CR3-IR activated microglia/macrophages, and GFAP-IR activated astrocytes primarily in the injection sites of rats received single injections of AAV9-hEPO, AAV9-Empty and PBS at both 4 days and 3 weeks (Fig. 5A–C). The median scores of arbitrary ratings for both CR3 and GFAP immunostaining were 3 among 4. These numbers were significantly reduced at 6 months (rating scores: 1, * p<0.05), suggesting that intrastriatal injections of PBS, AAV9-hEPO, or AAV9-Empty only led to transient inflammatory and innate immune responses in the striatum.

In addition to examining innate immune responses, we evaluated adaptive immune responses in the striatum by immunohistochemical staining for MHC class I, MHC class II, CD4, CD8 antigens in time course. MHC class I- and class II-IR cells resembled various leukocytes including macrophages and microglia. The immunostaining of endothelial cells in blood vessels that were uniformly stained by MHC class I antibody was used as an internal control for MHC class I immunostaining. There were dense MHC class I-immunostaining and large numbers of MHC class I-IR cells in the injected striatum 4 days and 3 weeks after single viral injections (rating scores: 3, Fig. 5A and B). Occasionally perivascular cuffing was present by rounded MHC class I- and class II-IR cells around blood vessels in the injected striatum. The levels of MHC class I antigen expression were significantly reduced 6 months after viral injections (rating scores: 1, * p<0.05). There were moderate levels of MHC class I-immunostaining and several patches of MHC class I-IR cells in the injected striatum 4 days and 3 weeks after single injections of PBS (rating scores: 2, Fig. 5C). The levels of MHC class I antigen expression were significantly reduced 6 months after single PBS injections (rating scores: 1, * p<0.05). The time pattern for the development of MHC class II immunostaining was different (Fig. 5A–C). At 4 days, a relatively low number of MHC class II-IR cells was observed in the injection sites of rats received single injections of AAV9-hEPO, AAV9-Empty, or PBS (rating scores: 1). At 3 weeks, the number of MHC class II-IR cells was significantly increased (rating scores: 2, * p<0.05). At 6 months, the number of MHC class II-IR cells was significantly reduced (rating scores: 1, * p<0.05).

Both CD4- and CD8-IR cells were easily distinguished with a red-brownish color against a pale background. Usually the immunoreactive cells were more abundant around the blood vessels near the needle tracks. Typically, the CD4-IR cells tended to be more weakly stained and were fewer in number than the CD8-IR cells. There seemed to be a correlation between the numbers of the two cell types. There were relatively low levels (the median scores: around 1) of infiltration of both CD4-IR and CD8-IR T cells in the injected striatum of rats received single injections of AAV9-hEPO and AAV9-Empty for three time-points (Fig. 5A and B). There was virtually no CD4- and CD8-immunostaining in the injected striatum of rats received single injections of PBS for three time-points (Fig. 5C).

For the dose-dependent study, there was in general a similar pattern of MHC class I-, MHC class II-, CR3- and GFAP-immunostaining in the injected striatum for all three doses of AAV9-hEPO. For inflammatory and innate immune responses, all three doses of AAV9-hEPO resulted in the large numbers of CR3-IR activated microglia/macrophages, and GFAP-IR activated astrocytes infiltrated in the injection sites 3 weeks after viral injections (Fig. 5D). Both CR3 and GFAP immunostaining were rated to 3. For adaptive immune responses, there were high levels of MHC class I and class II antigens in the injected striatum (the median scores: between 2 and 3). There was a trend in the increased numbers of CD4-IR T cells infiltrated in the injected striatum with increased doses of viruses. However, the difference in the number of CD4-IR T cells between different dose groups did not reach to a significant level. In contrast, the numbers of CD8-IR T cells in the intermediate and high dose groups appeared to be greater than that in the low dose group. Although the data did not show the dose-dependent effects of AAV9-hEPO on inflammatory and immune responses in the injected striatum, the levels of inflammatory and immune responses in the injected striatum were consistent with that in rats received single injections of AAV9-hEPO (with the same dose) 3 weeks after viral injections in the first experiment of the time course study of AAV9-hEPO single administrations.

**Figure 6:**
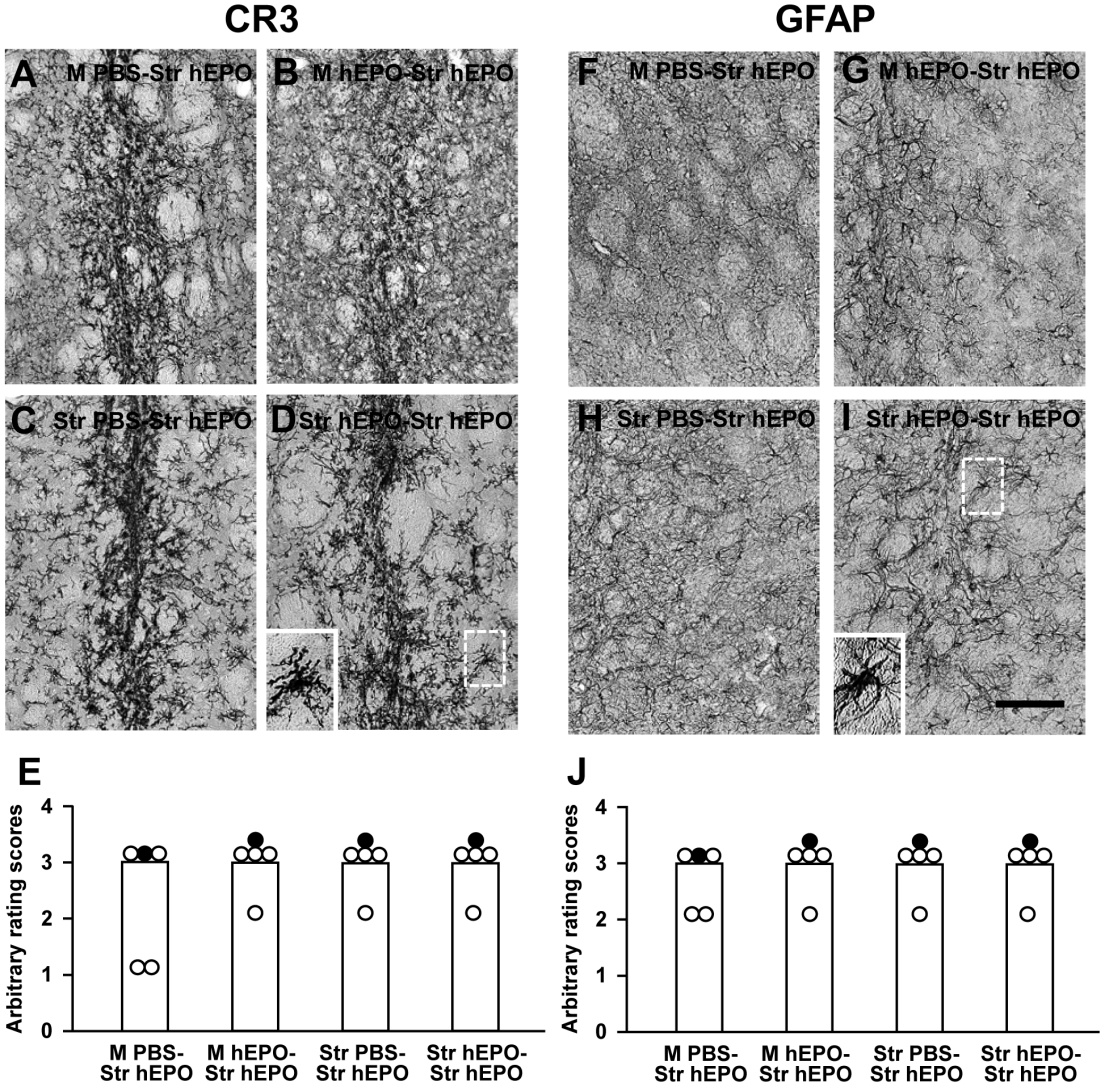
Accumulation of activated complement receptors 3 (CR3)-immunoreactive (IR) microglia and glial fibrillary acidic protein (GFAP)-IR astrocytes in the right injected striatum. Photomicrographs were prepared from coronal sections through the striatum and processed for CR3 (A–D) and GFAP (F–I) immunocytochemistry in representative rats of the M PBS-Str hEPO (A and F), M hEPO-Str hEPO (B and G), Str PBS-Str hEPO (C and H) and Str hEPO-Str hEPO (D and I) groups 3 weeks after the second injections. The scale bar = 100 µm in I also for A–D and F–H. High magnification photomicrographs in the left bottom were prepared from the box areas in (D) and (I). (E) and (J) Arbitrary rating scores of CR3 (E) and GFAP (J) immunostaining in the right injected striatum of rats from the M PBS-Str hEPO, M hEPO-Str hEPO, Str PBS-Str hEPO and Str hEPO-Str hEPO groups. The bars represent the median values of rating scores, and the circles depict the individual values. Filled circles indicate the rats from which the photomicrographs were taken.

**Figure 7:**
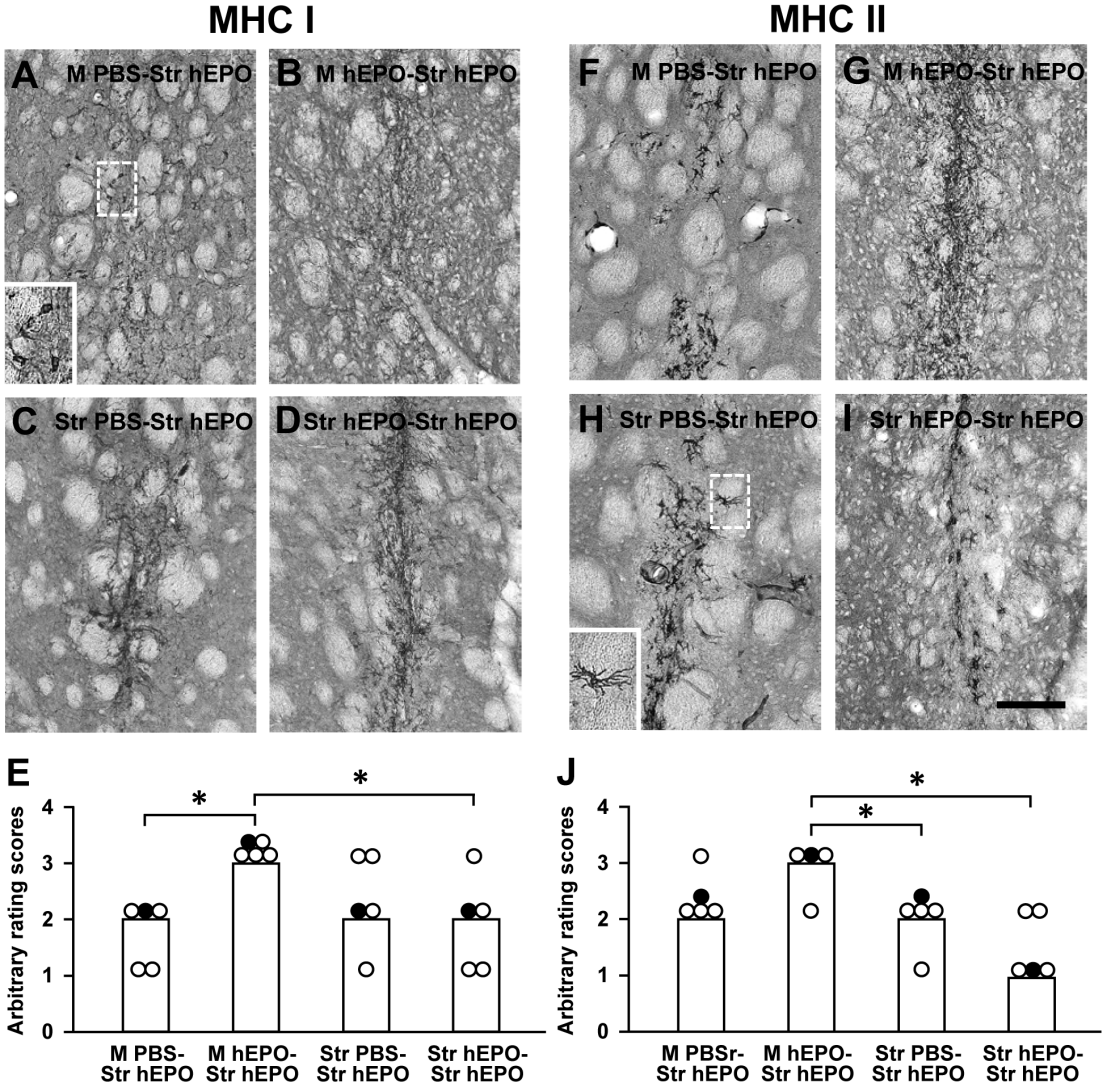
Expression of major histocompatibility complex (MHC) class I and class II antigens in the right injected striatum. Photomicrographs were prepared from coronal sections through the striatum and processed for MHC class I (A–D) and MHC class II (F–I) immunocytochemistry in representative rats of the M PBS-Str hEPO (A and F), M hEPO-Str hEPO (B and G), Str PBS-Str hEPO (C and H) and Str hEPO-Str hEPO (D and I) groups 3 weeks after the second injections. The scale bar = 100 µm in I also for A–D and F–H. High magnification photomicrographs in the left bottom were prepared from the box areas in (A) and (H). (E) and (J) Arbitrary rating scores of MHC class I (E) and MHC class II (J) immunostaining in the right injected striatum of rats from the M PBS-Str hEPO, M hEPO-Str hEPO, Str PBS-Str hEPO and Str hEPO-Str hEPO groups. The bars represent the median values of rating scores, and the circles depict the individual values. Filled circles indicate the rats from which the photomicrographs were taken. A Kruskal-Wallis test followed by Mann-Whitney U tests was used to compare the rating scores between groups. * p<0.05 versus indicated groups.

**Figure 8:**
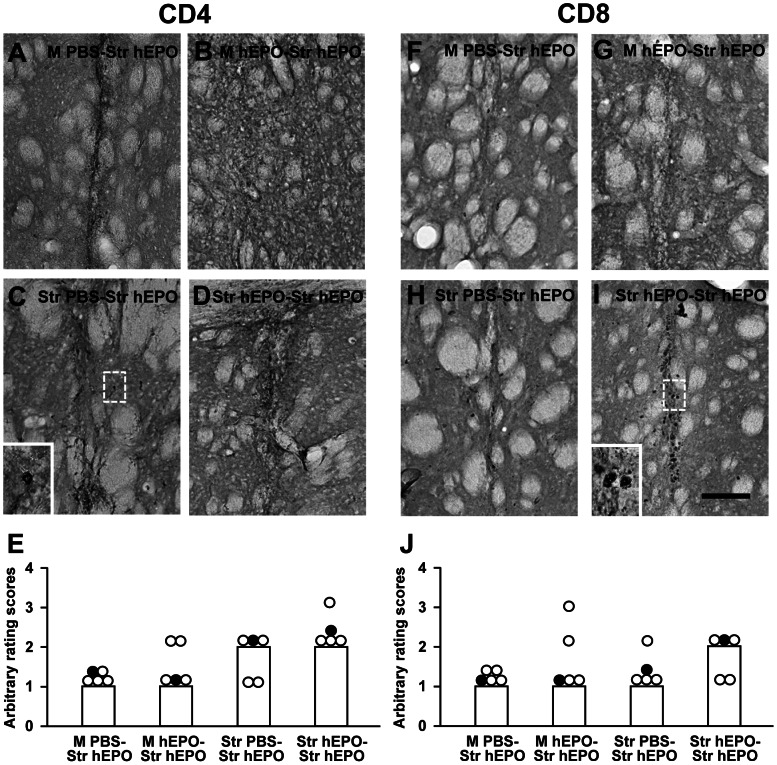
Infiltration of cluster of differentiation 4 (CD4)-immunoreactive (IR) and CD8-IR T cells in the right injected striatum. Photomicrographs were prepared from coronal sections through the striatum and processed for CD4 (A–D) and CD8 (F–I) immunocytochemistry in representative rats of the M PBS-Str hEPO (A and F), M hEPO-Str hEPO (B and G), Str PBS-Str hEPO (C and H) and Str hEPO-Str hEPO (D and I) groups 3 weeks after the second injections. The scale bar = 100 µm in I also for A–D and F–H. High magnification photomicrographs in the left bottom were prepared from the box areas in (C) and (I). (E) and (J) Arbitrary rating scores of CD4 (E) and CD8 (J) immunostaining in the right injected striatum of rats from the M PBS-Str hEPO, M hEPO-Str hEPO, Str PBS-Str hEPO and Str hEPO-Str hEPO groups. The bars represent the median values of rating scores, and the circles depict the individual values. Filled circles indicate the rats from which the photomicrographs were taken.

AAV9-hEPO re-administration resulted in the large numbers of CR3-IR activated microglia/macrophages (Fig. 6A–E), and GFAP-IR activated astrocytes (Fig. 6F–J) accumulation in the injection sites 3 weeks after second viral injections for all four groups. CR3-IR activated microglia/macrophages, and GFAP-IR activated astrocytes became more intensive along the needle tracks. The median scores for both CR3 and GFAP immunostaining were 3 (Fig. 6E and J). The similar pattern of CR3-IR activated microglia/macrophages, and GFAP-IR activated astrocytes accumulation was observed in the M PBS-Str hEPO group when compared to that in the other three groups (the M hEPO-Str hEPO, Str PBS-Str hEPO, and Str hEPO-Str hEPO). It suggests that intrastriatal injections of AAV9-hEPO can result in a certain extent of inflammation. For adaptive immune responses, the expression of MHC class I (Fig. 7A–E) and class II (Fig. 7F–J) antigens was pronounced in the M hEPO-Str hEPO group when compared to other three groups. A large number of MHC class I- and class II-IR cells were spread out needle tracks in the injected striatum. The median scores of arbitrary ratings were 3 for both MHC class I and class II immunostaining (Fig. 7E and J). The number of MHC class I-IR cells in the right striatum was greater in the M hEPO-Str hEPO group than that in the M PBS-Str hEPO and Str hEPO-Str hEPO groups (* p<0.05). The number of MHC class II-IR cells in the right striatum was greater in the M hEPO-Str hEPO group than that in the Str PBS-Str hEPO and Str hEPO-Str hEPO groups (* p<0.05). There were generally low numbers of CD4- (Fig. 8A–E) and CD8-IR (Fig. 8F–J) T cells infiltrating in the right striatum for all the four groups. No significant difference was observed for the median scores of arbitrary rating for CD4- and CD8-immunostaining among the groups (Fig. 8E and J, p>0.05). To illustrate the correlation between hEPO transduction, and inflammatory and immune responses, hEPO transduction, and inflammatory and immune responses in re-administration groups were summarized in Table 1.

**Table 1:** Summary of hEPO transduction and immune responses in the re-administration groups.

Groups	No. of hEPO^+^ cells	CR3^+^	GFAP^+^	MHC I^+^	MHC II^+^	CD4^+^	CD8^+^
M PBS-Str hEPO	630198±49820	3	3	2	2	1	1
M hEPO-Str hEPO	381673±63253*	3	3	3	3	1	1
Str PBS-Str hEPO	601780±78766	3	3	2	2	2	1
Str hEPO-Str hEPO	510015±51876	3	3	2	1	2	2

Note: The mean values of arbitrary rating are shown for CR3, GFAP, MHC class I and class II, and CD4 and CD8 immunocytochemistry. * p<0.05 versus the M PBS-Str hEPO and Str PBS-Str hEPO groups, one-factor analysis of variance (ANOVA) followed by Fisher's *post hoc* test.

### The levels of anti-AAV9 capsid protein and anti-hEPO antibodies in serum

**Figure 9:**
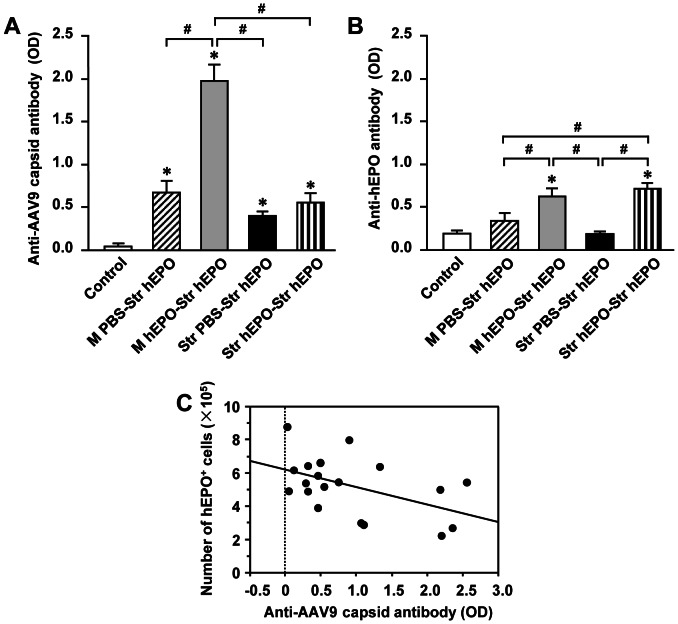
The levels of anti-recombinant adeno-associated virus serotype 9 (AAV9) capsid (A) and anti-human erythropoietin (hEPO) (B) antibody in sera of rats received AAV9-hEPO re-administration were detected by enzyme-linked immunosorbent assay (ELISA) for the Control (age matched normal rats, open bar, n = 5), M PBS-Str hEPO (hatched bar, n = 5), M hEPO-Str hEPO (gray bar, n = 5), Str PBS-Str hEPO (black bar, n = 5) and Str hEPO-Str hEPO (striped bar, n = 5) groups. The data are presented as mean values ± standard error of the mean (SEM). A one-factor analysis of variance (ANOVA) followed by Fisher's *post hoc* test was applied to make group comparisons. * p<0.05 versus the Control group; # p<0.05 versus the indicated groups. (C) Correlation between the total numbers(×10^5^) of hEPO-IR cells in the right injected striatum of rats in the M PBS-Str hEPO, M hEPO-Str hEPO, Str PBS-Str hEPO and Str hEPO-Str hEPO groups, and the levels of anti-AAV9 capsid antibody in their sera. Total numbers of hEPO-IR cells were plotted against the values of optical density for anti-AAV9 capsid antibody from three independent experiments. Dots represent the individual values. Total numbers of hEPO-IR cells in the right injected striatum were found to be negatively correlated with the values of optical density for anti-AAV9 capsid antibody from three independent experiments (r = 0.50, p<0.05). M, muscular; PBS, phosphate buffered saline; Str, striatal.

As expected, there were almost no detectable levels of anti-AAV9 capsid protein antibodies in the sera from control rats (the mean value of optical density: 0.04±0.04). In contrast, all the rats which received AAV9-hEPO developed a humoral response to AAV9 capsid protein by 3 weeks, regardless of the injection approaches (Fig. 9A, * p<0.05). The sera from the M hEPO-Str hEPO contained the greatest levels of anti-AAV9 capsid protein antibodies than other three groups (1.98±0.19, # p<0.05). The sera from the rats in the M PBS-Str hEPO, Str PBS-Str hEPO and Str hEPO-Str hEPO had moderate levels of anti-AAV9 capsid protein antibodies, which were significant greater than that in the control group (0.67±0.13, 0.40±0.05, and 0.55±0.12, respectively, * p<0.05 versus the Control). There was no significant difference in the levels of anti-AAV9 capsid protein antibodies among these groups (p>0.05). These results suggest that routes of administration determine the levels of a humoral response to AAV9.

Because the hEPO gene used in our study is xenogeneic to treated rats, we hypothesized that the products of hEPO gene could also elicit the humoral response in the hosts. The sera of the rats in the Control, M PBS-Str hEPO, M hEPO-Str hEPO, Str PBS-Str hEPO and Str hEPO-Str hEPO groups were also analyzed using ELISA for the levels of antibodies against hEPO (Fig. 9B). There were detectable levels of antibodies against hEPO in the sera from rats in the M hEPO-Str hEPO (0.57±0.13) and Str hEPO-Str hEPO (0.74±0.11) groups, which were significant greater than that in the Control (0.17±0.06) group (* p<0.05). The levels of antibodies against hEPO in the sera from rats in both the M hEPO-Str hEPO (0.57±0.13) and Str hEPO-Str hEPO (0.74±0.11) groups were also greater than that in the M PBS-Str hEPO (0.37±0.12) and Str PBS-Str hEPO (0.16±0.05) groups (# p<0.05).

To examine a correlation between the levels of anti-AAV9 capsid protein or anti-hEPO antibodies in the sera and hEPO transgene expression in the right striatum, all the individual values of the optical density indicating the levels of antibodies were plotted against all the individual values of unbiased stereological cell population estimation for the total numbers of hEPO-IR cells in the right striatum. The linear regression analysis showed that there was a negative correlation between the levels of anti-AAV9 capsid protein antibodies and the numbers of hEPO-IR cells in the striatum (Fig. 9C, r = 0.50, p<0.05).

### Effects of neutralizing antibody on transgene transduction

**Figure 10:**
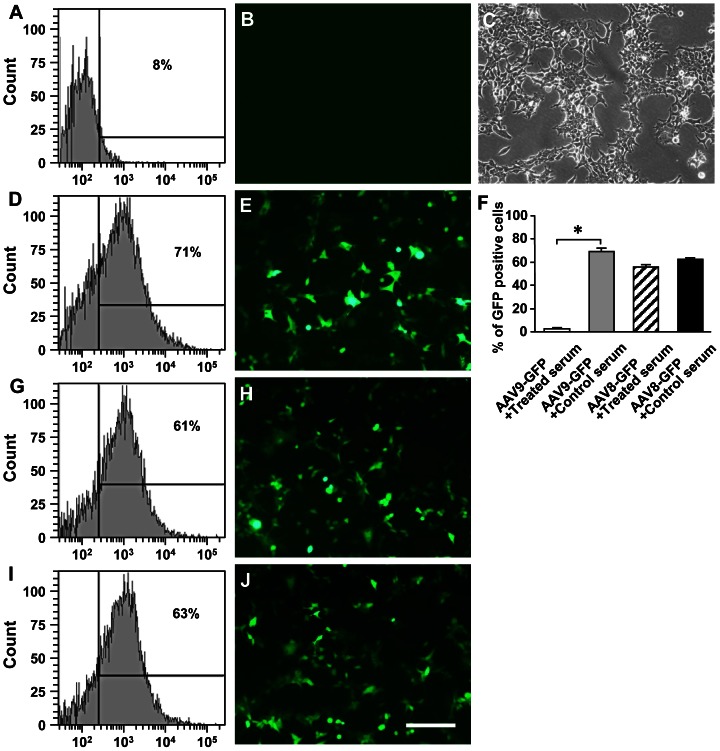
Immunoneutralization assay showing effects of sera from rats in the M hEPO-Str hEPO group on recombinant adeno-associated virus serotype 9 (AAV9)-or AAV8-mediated green fluorescent protein (GFP) transgene transduction in HEK293T cells. The sera from five rats in the M hEPO-Str hEPO group in which rats received the first injections of AAV9-hEPO into the right quadriceps and the second injections of AAV9-hEPO into the right striatum 3 weeks later were collected 3 weeks after the second injections. The control sera from five age matched normal rats were also collected. 25 µl treated or control sera was added to 2 µl of virus (AAV9-GFP or AAV8-GFP, 1.0×10^13^ vg ml^−1^) and directly incubated on HEK293T cells for transduction. Photomicrographs were prepared using a fluorescent microscope from HEK293T cell cultures treated with AAV9-GFP +treatedsera (B), AAV9-GFP+control sera (E), AAV8-GFP+treated sera (H), or AAV8-GFP+control sera (J). (C) A phase contrast photomicrograph was prepared from the same field of (B). The scale bar = 100 µm in J also for B, C, E and H. HEK293T cells (1.0×10^6^ ml^−1^) were also harvested from different treated cultures for flow cytometry analyses (A, D, G and I, representative flow cytometry charts). Note that treated sera blocked AAV9-mediated GFP transduction (A and B) but not AAV8-mediated GFP transduction on HEK293T cells (G and H). Incubation of the control sera had no effect on both AAV9- (D and E) and AAV8- (I and J) mediated GFP transduction. (F) Flow cytometry data from the AAV9-GFP + treated sera (open bar), AAV9-GFP + control sera (gray bar), AAV8-GFP + treated sera (hatched bar), and AAV8-GFP + control sera (black bar) treated cultures were summarized in the bar diagram. The data are presented as mean values ± standard error of the mean (SEM). A one-factor analysis of variance (ANOVA) followed by Fisher's *post hoc* test was applied to make group comparisons. * p<0.05 versus the indicated groups.

To assess whether circulating neutralization antibodies against AAV9 capsid or AAV9-mediated hEPO transgene product in serum contribute to reduction or inhibition of AAV9-mediated hEPO transduction after striatal re-administration, the sera were collected from the rats in the M hEPO-Str hEPO group and incubated with viruses prior to viral transduction on human embryonic kidney 293T (HEK293T) cells in vitro. It is known that neutralizing antibodies can bind to virus to prevent virus attachment to the cellular receptors and uptake into cells, leading to reduction of viral transduction. Our data show that sera from the rats in the M hEPO-Str hEPO group significantly reduced AAV9-mediated green fluorescent protein (GFP) transduction on HEK293T cells (3.2±0.9%) when compared with the control (68.8±3.3%), as assessed by FCM and an inverted fluorescent microscope (Fig. 10A–C, F, * p<0.05). A representative FCM data showed that the proportion of GFP-positive cells only accounted for 8% in the total cell population (Fig. 10A), and GFP-positive cells were not detected in a particular microscopic field (Fig. 10B) which contained numerous cells in a phase contrast image (Fig. 10C). To determine whether the effects of neutralizing antibody on transgene expression are viral serotype (AAV9) specific, sera from the rats in the M hEPO-Str hEPO group were incubated with AAV8-GFP viruses prior to adding to HEK293T cell cultures. This treatment did not result in reduction of AAV8-mediated GFP transduction (52.1±2.4%) when compared to the control (62.6±0.7%) (Fig. 10G, H, F, I, and J, p>0.05), indicating the presence of AAV9 capsid-specific antibodies in the sera of rats in the M hEPO-Str hEPO group. Incubation of sera from naïve rats (age matched normal rats) with each viruses (AAV9-GFP or AAV8-GFP) was used as controls, and did not reduce the corresponding viral transduction (Fig. 10D, E, F, I, and J), suggesting the lack of cross reactivity.

### Effects of the hEPO gene delivery on erythropoiesis

**Figure 11:**
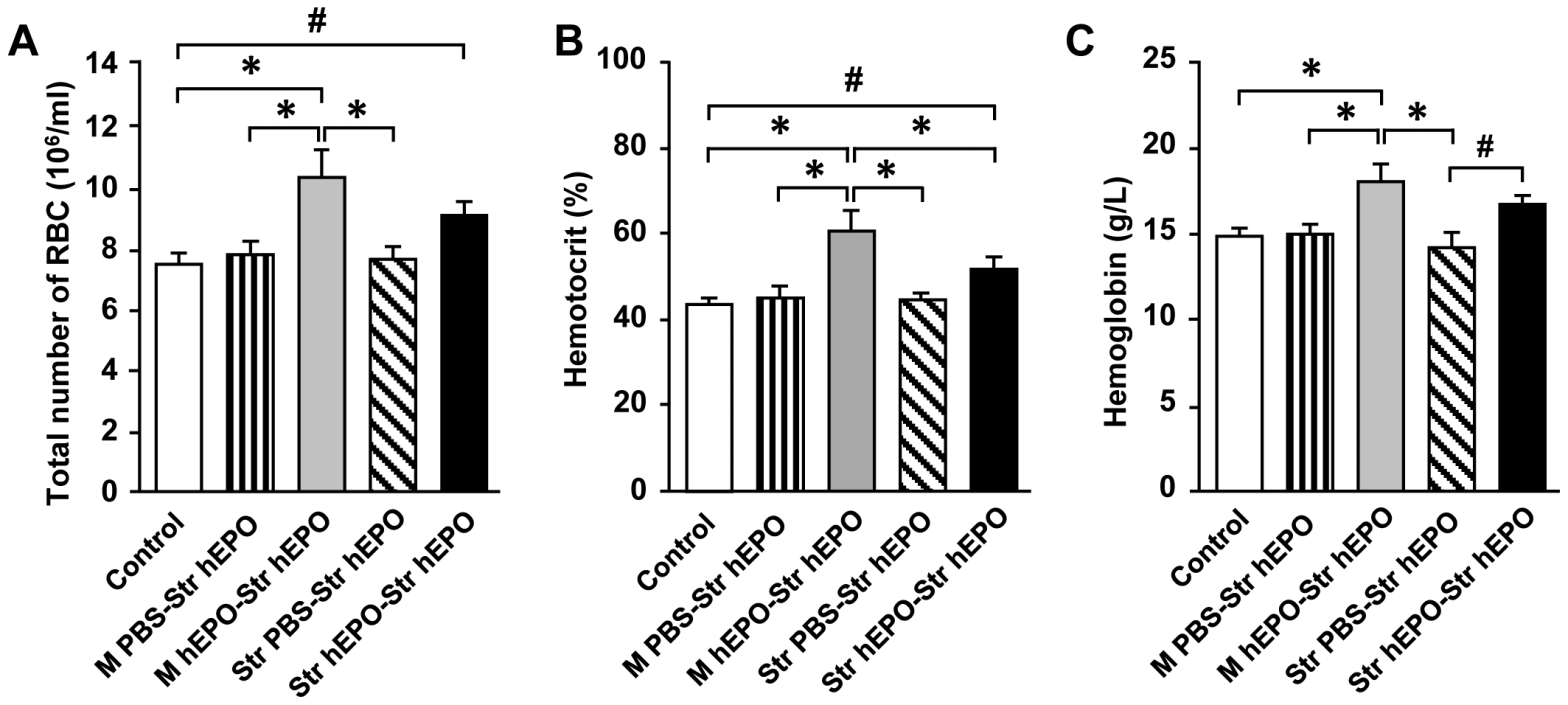
Effect of recombinant adeno-associated virus serotype 9-human erythropoietin (AAV9-hEPO) re-administration on erythropoiesis. Peripheral blood samples were collected 3 weeks after the second injections of AAV9-hEPO into the right striatum for hematological assays. The bar diagrams summarizes: (A) The number of red blood cells(×10^6^ ml^−1^), (B) hemotocrit (%) of peripheral blood, and (C) hemoglobin (g/l) in peripheral blood per rat in the control (age matched normal rats, open bar, n = 5), M PBS-Str hEPO (striped bar, n = 5), M hEPO-Str hEPO (gray bar, n = 5), Str PBS-Str hEPO (hatched bar, n = 5) and Str hEPO-Str hEPO (black bar, n = 5) groups. The data are presented as mean values±standard error of the mean (SEM). A one-factor analysis of variance (ANOVA) followed by Fisher's *post hoc* test was applied to make group comparisons. *, # p<0.05 versus the indicated groups. M, muscular; PBS, phosphate buffered saline; Str, striatal.

To examine whether the hEPO gene delivery into the muscle or into the brain could have systemic effects on erythropoiesis, the number of red blood cells (RBC), hematocrit and hemoglobin were measured in peripheral blood. The number of RBC (10^6^ ml^−1^) was significantly greater for the rats in the M hEPO-Str hEPO group (10.4±0.9, Fig. 11A, * p<0.05) than that in the Control (7.5±0.3), M PBS-Str hEPO (7.9±0.4), and Str PBS-Str hEPO (7.9±0.3). The number of RBC (10^6^ ml^−1^) was also significantly greater for the rats in the Str hEPO-Str hEPO group (9.1±0.4, # p<0.05) than that in the Control (7.5±0.3). The levels (%) of hematocrit were significantly greater for the rats in the M hEPO-Str hEPO group (60.2±5.4, Fig. 11B, * p<0.05) than that in the Control (42.8±1.6), M PBS-Str hEPO (45.9±2.3), Str PBS-Str hEPO (44.6±1.8), and Str hEPO-Str hEPO (51.5±2.3). The levels (%) of hematocrit were also significantly greater for the rats in the Str hEPO-Str hEPO (51.5±2.3, # p<0.05) than that in the Control (42.8±1.6). The levels (gram/L) of hemoglobin were significantly greater for the rats in the M hEPO-Str hEPO group (18.7±1.1, Fig. 11C, * p<0.05) than that in the control (15.0±0.5), M PBS-Str hEPO (15.2±0.6), and Str PBS-Str hEPO (14.5±0.9). The levels (%) of hematocrit were also significantly greater for the rats in the Str hEPO-Str hEPO (17.1±0.5, # p<0.05) than that in the Str PBS-Str hEPO (14.5±0.9).

## Discussion

In the present study, we used AAV9-mediated hEPO gene transfer into the rat brain as a model system to systematically characterize inflammatory and immune responses to intrastriatal AAV9-hEPO vectors when recipient rats were at different immune status with intrastriatal or intramuscular injections of AAV9-hEPO vectors. In consistent with the observations in the previous study, we show that AAV9 vectors can efficiently deliver the hEPO gene into the rat brain leading to a robust and prolonged hEPO transgene expression for 6 months. Intrastriatal injections of AAV9-hEPO trigger inflammatory and immune responses to the vectors in the brain as evaluated by the levels of MHC class I and class II antigen expression, infiltration of CD4- and CD8-lymphacytes, and accumulation of activated microglial cells and astrocytes. The hEPO transgene expression exhibits in a time course and dose dependent manner, and inflammatory and immune responses display in a time course manner. For single striatal administration of AAV9-hEPO vectors, the levels of inflammatory and immune responses subside following the time. The first intrastriatal injections of AAV9-hEPO do not lead to reduction of the vector-mediated transgene expression following striatal re-administration. In contrast, intramuscular injections of AAV9-hEPO result in reduced levels of hEPO transduction and increased levels of inflammatory and immune responses in injected striatum. Moreover, the sera from the rats with intramuscular injections of AAV9-hEPO contain greater levels of antibodies against both AAV9 capsid protein and hEPO protein than that in the other treatment groups. hEPO gene expression appears to be negatively correlated with the levels of circulating antibodies against AAV9 capsid protein. In addition, both intramuscular injections of AAV9-hEPO and intrastriatal re-administration of AAV9-hEPO result in increased numbers of red blood cells in peripheral blood.

Several studies have been performed to examine inflammatory and immune responses to AAV vectors-mediated hAADC [17], [25], GFP and GDNF [21], [22], beta-galactosidase [31], firefly luciferase [32] gene transfer into the brain. However, inflammatory and immune responses to AAV9 vectors-mediated hEPO gene transfer to the brain have not yet been determined. Toxicity or instability of transgene products has emerged as an obstacle for the studies. Ciesielska et al. showed that intrastriatal injections of AAV9-hAADC resulted in a significant loss of neurons and induction of anti-hAADC antibodies [17]. By using striatal re-administration experimental paradigm, Peden et al. demonstrated that striatal AAV2-mediated GDNF gene transduction remained unaffected while AAV2-mediated GFP gene transduction was severely reduced in the second-injection sites [22]. GDNF is a very stable protein in the brain that allows the accumulation in the brain over time. GFP can also accumulate inside cells, but is often toxic to cells. A previous study showed that the high levels of GFP delivered by AAV8 vectors were neurotoxic to DA neurons in the SN [33]. Firefly luciferase is not a stable protein in the brain. Indeed, Mastakov et al. demonstrated that a reduction of striatal transgene expression in the second-injection site following striatal re-administration by using AAV carrying the firefly luciferase gene [32]. In the current study, we extend our previous observation of a long-term AAV9-mediated hEPO transgene expression from 10 weeks to 6 months. It is worthy of note that AAV9-mediated hEPO transduction reached to a full scale level at 3 weeks in our time course study, suggesting it needs a time for hEPO transgene to fully express and for hEPO transgene product to accumulate. However, Reimsnider et al. showed that AAV-mediated GFP transduction reached to maximal striatal cell GFP-IR cell counts at 4 days after injection as examined by GFP immunohistochemical staining [34]. The discrepancies in the results may be due in part to the methods of viral preparation, viral administration, and transgene product. At 6 months after transduction, the number of hEPO-IR cells in the striatum is not different from that at 3 weeks, suggesting that AAV9-mediated hEPO transduction is stable over time. Although hEPO gene product is xenogeneic to recipient rats, AAV9-mediated hEPO gene transfer into the rat brain can only cause transient inflammatory and immune responses which allow a long-term hEPO transgene expression. This observation is in agreement with the previous finding that the inflammatory response to AAV vectors was only present up to 4 weeks after transduction but was not apparent 9 months post injection [34]. In the current study, striatal administration of AAV9-hEPO exhibited a dose-dependent increase of hEPO-IR cells in the striatum. It is interesting to note that increased number of hEPO-IR striatal cells does not accompany with increased levels of inflammatory and immune responses in the virally injected striatum. The results are contrary to our hypothesis of a positive correlation between the number of transduced cells and the levels of inflammatory and immune responses. We speculate that the lowest dose (5.9×10^11^ vg ml^−1^) of AAV9-hEPO used in the study is already very high. The levels of inflammatory and immune responses may stay at a plateau stage under this dose of AAV9 vectors. Additional study is warranted to examine changes in the levels of inflammatory and immune responses with a series of doses of AAV9-hEPO below the dose of 5.9×10^11^ vg ml^−1^. It has been suggested that EPO possesses multiple functions including anti-apoptosis [35], [36], anti-inflammation [37], inhibition of glutamate release and reactive oxygen species formation [38], activation of Akt/protein kinase B via the phosphoinositide 3-kinase pathway [35], and activation of Janus kinase-2 and nuclear factor-kappa B signaling pathways [39]. In a recent study, we also demonstrated that intrastriatal administration of EPO reduced inflammation in the substantia nigra ipsilateral to 6-OHDA-lesioned striatum [40]. The property of anti-inflammation for hEPO may attribute to the stability of hEPO protein in the brain. Our study provides a good model system to carefully examine the nature of inflammatory and immune responses to AAV9-mediated gene transfer into the rat brain.

The brain has long been considered an immunologically privileged site, due to the existence of the BBB, absence and paucity of APCs, and lack of lymphatic drainage system. Because the brain has two hemispheres, it allows doing striatal re-administration of neural transplant, pharmacological agents or virus vectors to examine the effect of the first striatal intervention in one hemisphere on the second striatal intervention in the other hemisphere. In a previous study, we performed sequential neural transplantation to examine the effects of immune response induced by the first neural allografts on the survival of the second striatal neural allografts [41]. Although the first neural allografts in the left striatum increased the levels of immune response against the second neural allografts in the right striatum as measured by the expression of MHC class I and II antigen and accumulation of activated microglia in the striatum, the survival of the second neural allografts was not impaired, confirming the property of immunological privilege for the brain [41]. However, accumulating evidence has showed that the immunological privilege of the brain is relative and can be abrogated by a strong immune stimulus, for example, intrastriatal neural xenotransplantation, addition of allogeneic spleen cells into the neural allografts, and pre-immunization with skin allografts [42]–[45]. Experimental paradigm of striatal re-administration has also been used to examine the basic brain immune response to AAV, and the results are conflicting [21], [22], [31], [32]. Differences in methods of viral administration and transgene products may attribute to these discrepancies. Since the BBB plays an important role in the properties of immunological privilege for the brain, the different degree of disruption to the BBB during the injections of AAV vectors into the brain among animals has been accounted for a cause of inconsistent results for striatal re-administration of AAV vectors carrying the beta-galactosidase gene [31]. To increase the opening of the BBB and an opportunity of immunization, Mastakov et al. injected a mixture of AAV and mannitol into the striatum in a striatal re-administration study [32]. The results from the current study have extended and supported our previous observations of striatal re-transplantation of neural allografts to striatal re-administration of AAV9-hEPO. Single striatal injections of AAV9-hEPO only resulted in transient inflammatory and immune responses in the injected striatum. The first injections of AAV9-hEPO in the left striatum did not lead to a reduction of AAV9-mediated hEPO transgene expression after repeating administration. In contrast, intramuscular injections of AAV9-hEPO efficiently immunized rats, and led to reduction of AAV9-mediated hEPO transgene expression following the sequential injections into the striatum. These data further suggest that the immunological privilege of the brain is not complete.

Our results have extended the previous observations that AAV-mediated gene transfer into the brain can trigger humoral and cellular immune responses [16]–[26]. Innate responses are the first line of defense in viral infection to block or inhibit viral infection. In our study, activated microglia and astrocyte were detected in the virally injected striatum, suggesting that AAV9-mediated hEPO gene transduction does elicit the innate immune response. Expression of MHC class I and class II antigens, and infiltration of CD4- or CD8-IR T cells against AAV9-hEPO vectors are components of adaptive immune processes. Our results show that AAV-mediated gene transfer into the brain initiates adaptive immune processes as measured by expression of MHC class I and class II antigens, and infiltration of CD4- or CD8-IR T cells. MHC molecules play a key role in determining whether virus transfected cells will be accepted as self or rejected as foreign, and presenting to cytotoxic or helper T cells. We observed increased levels of MHC class I and MHC class II antigen expression in the injected striatum following striatal re-administration. MHC class I- and class II-IR cells resembled microglia which may function as APCs. By using double immunofluorescence staining, Ciesielska et al. recently reported that AAV9-hAADC vectors, when injected into the rat striatum, were capable of transducing APCs in the striatum as many of the MHC class II-IR cells also expressed Iba1, a microglial marker, and provoking a cell-mediated immune response [17]. The infiltration of CD8-IR T cells in the rat striatum raises the possibility that AAV9 transduced cells are killed. However, we did not observe the significant levels of CD8-IR T cell infiltration and a significant cell loss in the striatum. Thus, the cell-mediated immunity may play a less role in the loss of striatal hEPO transgene expression in our model system. Indeed, several studies have reported that AAV2 capsid specific cell-mediated immunity does not appear to play a role in elimination of AAV-transduced cells in mouse models [22], [46], [47].

It has been demonstrated that neutralizing antibody produced in the first administration prevented the second application. Because wild type AAV does not ordinarily infect rodents, rodents are naïve to wild type AAV and normally lack pre-existing neutralizing antibodies and helper T cells to recognize AAV vectors. Clinical studies have shown that the translations of AAV-mediated gene therapy into humans unexpectedly result in only short-term expression of the therapeutic. The vast majority of human population has been exposed to wild type AAV. To mimic the anti-AAV9 immunization status of patients in clinic, we sensitized rat immune system with intramuscular or intrastriatal injections of AAV9-hEPO. Injections of AAV9-hEPO intramuscularly led to a high production of anti-AAV9 antibody, whereas, intrastriatal injections resulted in minimal anti-AAV9 neutralization antibody production. As a result, hEPO transgene expression was reduced in the striatum of rats in the M hEPO-Str hEPO group. hEPO transduction was negatively correlated with the levels of neutralizing antibodies against AAV9 capsid protein. Several studies have shown that a humoral immune response against AAV vectors plays a more important role in preventing re-administration of AAV vectors rather than transgene product-induced immune response [21], [23], [24]. Our *in vitro* study further supports this notion. Anti-AAV9 capsid antibody in serum completely blocks AAV9-mediated GFP transgene expression in HEK293T cells. However, anti-AAV9 capsid antibody in serum did not block AAV8-mediated GFP transgene expression HEK293T cells. These findings suggest that the neutralizing antibodies against AAV9-hEPO are specific to the viral antigens rather than the transgene product.

In the present study, AAV9-mediated EPO gene transfer into both the muscle and the brain of the rats in the M hEPO-Str hEPO group exhibited clear systemic effects to enhance erythropoiesis as measured by total number of red blood cells, hematocrit and hemoglobin in peripheral blood, suggesting the vector derived EPO was biologically active. Repeated injections of AAV9-hEPO vectors into the striatum of the rats in the Str hEPO-Str hEPO group also had enhanced hematopoietic activities. It is noted that these systemic effects did not appear in the rats of the M PBS-Str hEPO and Str PBS-Str hEPO groups, which received single injections of AAV9-hEPO in the striatum. This observation was inconsistent with that in our previous study [12]. The discrepancies in the results may be due in part to the methods of viral injection, blood collection, and hematologic examination. In a previous study, we speculated that EPO encoded by the EPO gene in the striatum could pass through the BBB into the circulation, stimulating hematopoietic stem cells in bone marrow to produce more red blood cells in peripheral blood [12]. The possible side effects of EPO expression highlight the importance of using a mutated EPO gene encoding non-hematopoietic EPO and introducing an EPO gene regulatory system.

In summary, we systematically characterize inflammatory and immune response to AAV9-mediated hEPO gene transfer into the striatum of rats which were at different immune status. Our findings indicate that intrastriatal injections of AAV9-hEPO can immunize rats. Although the levels of immunization subside following time, it should be cautious that when an increased dose of virus is applied to enhance therapeutic efficacy, virus-induced inflammatory and immune responses must also be considered. In addition, the intrastriatal injections of AAV9-hEPO do not result in reduction of hEPO transduction in striatal re-administration, suggesting that sequential administration of the same vectors can be tolerated in the rat brain. However, systemic administration of AAV9-hEPO with intramuscular injections can efficiently immunize rats, leading to reduction of AAV9-mediated hEPO transgene expression in the rat brain. It suggests that primed immune system can jeopardize AAV9-mediated gene transfer into the brain.

## Materials and Methods

### Subjects and experimental design

A total of 82 young adult female Sprague-Dawley rats, weighing 225–250 g at the beginning of this experiment, were obtained and housed under a 12 h light/dark cycle with *ad libitum* access to food and water in the Animal Core Facility of Capital Medical University (CMU), Beijing, China. All animal procedures were done following the National Institutes of Health Guide for the Care and Use of Laboratory Animals and were approved by the Animal Use and Care Committee of CMU. The number of animals used was the minimize animal required for statistical analysis, and all precautions were taken to minimize animal suffering. The rats were assigned randomly into treatment groups shown schematically in Figure 1. The treatment regimens, time of survival of each group, and number of rats per group are presented in Figure 1. The time course (Fig. 1A) and dose dependent (Fig. 1B) experiments were designed to establish the base line of immune and inflammatory responses in the striatum after single intrastriatal injections of PBS, AAV9-hEPO and AAV9-Empty. AAV9-hEPO re-administration groups (Fig. 1C) were designed to examine immune and inflammatory responses in the right striatum of rats with different routes of immunization.

### DNA and virus production

The human EPO DNA was obtained from pCR4-TOPO vectors (ATCC, Manassas, VA, USA) and subcloned into an AAV expression plasmid using the EcoR1 enzyme. The expression cassette was flanked with AAV2 terminal repeats and cross-packaged into recombinant AAV9 or AAV8. The hybrid cytomegalovirus immediate early enhancer/chicken β-actin promoter was used to drive expression. AAV9 vectors for either hEPO, GFP or Empty (without a transgene), and AAV8 vectors for GFP were constructed as previously described [48]. HEK293T cells were co-transduced with the hEPO plasmid along with the two packaging plasmids needed to make AAV9. The cell lysate was applied to a discontinuous gradient of iodixanol (Opti-Prep, Greiner Bio-One, Longwood, FL, USA) and centrifuged. The AAV was then removed, diluted twofold with lactated Ringer's solution (Baxter, Deerfield, IL, USA) and then washed and concentrated by Millipore (Billerica, MA, USA) Biomax 100 Ultrafree-15 units. The final stocks were sterilized by Millipore Millex-GV syringe filters into low adhesion tubes (USA Scientific, Ocala, FL, USA). Vectors were aliquoted and stored frozen. Encapsidated genome copies were tittered by dot blot. The titer of the AAV9-hEPO was 5.9×10^13^ vector genomes (vg) ml^−1^, the AAV9-GFP was 1.0×10^13^ vg ml^−1^, and the AAV9-Empty was 5.9×10^13^ vg ml^−1^. Equal dose comparisons were made by normalizing titers with the diluent, PBS.

### Stereotaxic injections into the striatum and injections into the quadriceps

A total of 2 µl of viral vectors (AAV9-hEPO, 5.9×10^13^ vg ml^−1^) or sterile PBS were stereotaxically injected into either the left or right striatum of equithesin (3 ml kg^−1^, i.p.) anesthetized rats fixed in a Kopf stereotaxic frame using a 10 µl Hamilton microsyringe (Hamilton Co., Reno, NV, USA) fitted with a steel cannula. Injections were made at the following stereotaxic coordinates: 1.0 mm rostral to bregma; 3.0 mm lateral to the midline (left or right side); 4.5 mm ventral to the dura, with tooth bar set at zero. The microinjections were carried out at a rate of 0.25 µl min^−1^. For the dose dependent study, AAV9-hEPO was diluted in PBS into three different doses (5.9×10^11^, 5.9×10^12^, and 5.9×10^13^ vg ml^−1^) for striatal injections. After injection, the cannula remained in situ for an additional 4 min before being withdrawn. 200 µl of AAV9-hEPO (in a diluted titer of 5.9×10^11^ vg ml^−1^) or sterile PBS were slowly injected using 1 ml insulin syringes into the right quadriceps of equithesin (3 ml kg^−1^, i.p.) anesthetized rats.

### Immunocytochemistry

Rats were deeply anesthetized with equithesin (3 ml kg^−1^, i.p.) and transcardially perfused with 0.1 M PBS followed by cold 4% formaldehyde in PBS. The brains were then removed and post-fixed for 4 h in the same fixative, and placed in 20% sucrose in PBS at 4 °C until they sank. Brain sections were coronally cut through the striatum and SN at 30 µm thickness on a sliding microtome. Four sets of serial sections for each rat were collected into anti-freeze solution using a 24-well multi-well plate. The avidin–biotin complex immunoperoxidase technique was used to visualize hEPO, MHC class I, MHC class II, CD4, CD8, CR3 and GFAP immunoreactivity, as described previously [44]. The primary antibodies were rabbit anti-hEPO (1∶300, Santa Cruz Biotechnology Inc), mouse anti-MHC class I, class II, and CR3 (1∶400, MRC OX-18, OX-6 and OX-42, respectively, Serotec, Oxford, UK), and mouse anti-GFAP (1∶500, Sigma). The secondary antibodies were biotinylated goat anti-rabbit (rat-absorbed) (1∶200, Vector Laboratories, Burlingame, CA, USA). Brain sections were incubated in ABC solution (Vectastain ABC Elite kit, Vector Laboratories) followed by development with 3,3′-diaminobenzidine solution (Vectastain DAB kit, Vector Laboratories) to visualize immunoreactivity. Brain sections were then mounted on superfrost microscope slides (Fisher Scientific, Pittsburgh, PA, USA), dehydrated through ascending graded concentrations of alcohol, cleared in xylene and coverslipped using DPX mountant (Fluka, Buchs, Switzerland). To evaluate the specificity of immunostaining, normal mouse serum was used instead of a primary antibody on selected brain sections, or primary antibodies were omitted during the immunostaining as a negative control.

### Sterological cell counts

For morphological assessment, immunostained brain sections were examined using a Nikon light microscope (Nikon, Japan) with bright-field illumination in a blinded manner. The original coding of the slides, which indicated treatment groups, was covered by opaque tape and the slides were re-numbered. After evaluation, the original codes were revealed. Immunostained brain sections were examined using a Leica DM 4000B light microscope (Leica microsystems Ltd, Germany) with bright-field illumination in a blinded manner, and total number of hEPO-IR cells in the right injected striatum were examined using the Optical Fractionator probe for unbiased cell population estimates in Stereo Investigator Version 5 by MicroBrightField (the MicroBrightField Inc system, Williston, VT, USA), as described previously [49]. Optical dissectors were 50×50×19 µm cubes spaced in a systematic random manner 700×700 µm apart and offset 2 µm from the section surface for a total volume of 33250 µm^3^ for all rats in the study. The coefficient of error was<0.1 in each estimate.

### Semi-quantitative evaluation of immunostained brain sections

Right striatal sections immunocytochemically processed for MHC class I, MHC class II, CD4, CD8, CR3 and GFAP immunostaining were semi-quantitatively evaluated, as described previously [44]. For each rat and primary antibody, two-to-three sections containing the needle track were rated by two independent raters into one of the following categories: (0) no specific immunoreactive cells in the injected striatum; (1) a small number of immunoreactive cells distributed as scattered single cells or clustered in a few small patches in the injected striatum; (2) several immunoreactive cells distributed as single cells or clustered in multiple, prominent patches in the injected striatum; (3) dense immunostaining and a large number of immunoreactive cells in the injected striatum; (4) very dense immunostaining and a very large number of immunoreactive cells in the injected striatum. In general, the rating scores given by two raters were very similar. The final score for each rat was determined based on the highest score observed and the median score for each group was plotted. Cell morphology and level of immunostaining of microglia and astrocytes in the left uninjected striatum of rats from the time course and dose dependent study groups were used to define resting microglia and astrocytes. Both activated and resting cells were labeled by the CR3 and GFAP antibodies, but resting cells were smaller and less intensely stained than activated microglia and astrocytes in the injected striatum. Only activated microglia/macrophages and astrocytes were included in the rating.

### Blood collection and serum sample preparation

At the time of sacrifice, a group of age matched normal rats (n = 5) and the rats in the re-administration groups were anesthetized with equithesin (3 ml kg^−1^, i.p.), 0.3 ml of whole blood was collected from the external jugular vein using a 23-gauge needle on a 1 ml syringe, and placed into a BD Microtainer Tube with heparin (BD Biosciences, San Jose, CA, USA). Blood samples were examined for red blood cell counts, hematocrit and hemoglobin. The blood was allowed to coagulate on ice for 30 minutes and then centrifuged for 10 minutes at 6,000 rotations per minute. The serum was then extracted and stored at −80 °C for further antibody and immunoneutralization assays.

### Anti-AAV9 capsid protein and anti-hEPO antibody assays

The levels of anti-AAV9 capsid protein and anti-hEPO antibodies in serum were determined by enzyme-linked immunosorbent assay (ELISA). Ninety-six well Nunc MaxiSorp® flat-bottom 96 well plates (eBioscience, Inc. San Diego, CA, USA) were coated with 100 µl of PBS containing AAV9-hEPO (10^11^ vg ml^−1^) or 100 µl of PBS containing 2 units hEPO (EPOGEN, Amgen, Inc. Thousand Oaks, CA, USA) at 4°C overnight. The wells were then washed and incubated with 5% BSA in PBS for an hour at room temperature. To determine a proper dilution of sera for further assays, the sera from the rats were diluted in series (1∶40, 1∶400, 1∶4,000, 1∶40,000, 1∶400,000, 1∶4,000,000 for anti-AAV9 capsid protein assay, and 1∶50, 1∶100, 1∶200, 1∶400, 1∶800, 1∶1,600 for anti-hEPO antibody assay). 100 µl of diluted serum (1∶40000 for anti-AAV9 capsid protein assay and 1∶200 for anti-hEPO antibody assay) from the rats in the re-administration groups or from age matched normal rats were loaded into the wells at 4°C overnight. The plates were washed and incubated with 100 µl of 1∶5000 dilution of HRP-conjugated goat anti-rat IgG for an hour at room temperature, and then developed with TMB reagent (Fisher scientific, Pittsburgh, PA). The wells were loaded 100 µl of 1 M hydrogen chloride to stop development and read at 450 nm with a microplate reader. The values of anti-AAV9 capsid protein and anti-hEPO antibodies were presented as the optical density (OD) value at 450 nm. The data of ELISA were collected from three independent experiments.

### Antibody and immunoneutralization assay

HEK293T cells (2.0×10^5^ cells/well) were plated into 6-well plates and maintained in Dulbecco's modified Eagle's medium (DMEM) medium supplemented with 10% heat-inactivated fetal bovine serum (FBS) and 1% penicillin/streptomycin at 37 °C under an atmosphere of 5% CO_2_ and 95% air. The cells were used for further experiment when they reached to 70% confluence. The treated sera from rats in the M hEPO-Str hEPO group or control sera from age matched normal rats was exposed to 2 µl of AAV9-GFP (1.0×10^13^ vg ml^−1^) or AAV8-GFP (1.0×10^13^ vg ml^−1^) for 1 hour at 37°C. The mixture of the serum and AAV9-GFP or AAV8-GFP vectors was then added to HEK293T cell cultures for transduction. The medium was changed to normal medium without viruses 6 h later. After 48 h, viral transduction on HEK293T cells was examined with an inverted fluorescent microscope (Nikon, Japan) and fluorescent images were prepared. HEK293T cells (1×10^6^ ml^−1^) were also harvested for flow cytometric (FCM) analysis. Briefly, the treated HEK293T cells were digested with 0.25% trypsin +EDTA(Sigma) and centrifuged at 1000 rpm for 5 min, the supernatant was then removed. The cells were washed twice with PBS and adjusted to a concentration of 1×10^6^ cells/ml for FCM analysis. To determine the amount of serum necessary to neutralize the virus, 10 µl, 25 µl, or 50 µl of treated serum was added to 2 µl of virus. Addition of 10 µl of treated serum to 2 µl of virus was found not to change the transgene expression in this assay, suggesting that the amount of antibodies in the serum was not sufficient for neutralizing antibody formation. In contrast, addition of 25 µl of treated serum was observed to efficiently block viral transduction and therefore used in the following experiments. At least three replicas per group were used in each experiment. Data were collected from at least three independent experiments.

### Statistical analysis

Data are presented as means ± standard error of the mean (SEM). A one-factor ANOVA followed by Fisher's *post hoc* test was used to compare the hEPO positive cells number, the levels of neutralizing antibodies in serum, the percentage of GFP positive cells and the physiological analyses of blood cells. Non-parametrically using a Kruskal-Wallis test followed by Mann-Whitney U tests was used to compare the arbitrary rating scores between groups. Regression analysis was used to examine correlation between the total numbers of hEPO-IR cells in the right injected striatum of rats and the levels of anti-AAV9 capsid antibody in their sera. Statistical significance was defined at p<0.05.
